# Stratification of Patients with Diabetes Using Continuous Glucose Monitoring Profiles and Machine Learning

**DOI:** 10.34133/2022/9892340

**Published:** 2022-04-27

**Authors:** Yinan Mao, Kyle Xin Quan Tan, Augustin Seng, Peter Wong, Sue-Anne Toh, Alex R. Cook

**Affiliations:** ^1^Saw Swee Hock School of Public Health, National University of Singapore and National University Health System, Singapore; ^2^Department of Statistics and Data Science, National University of Singapore, Singapore; ^3^NOVI Health, Singapore; ^4^Yong Loo Lin School of Medicine, National University of Singapore, Singapore; ^5^Duke-NUS Medical School, Singapore

## Abstract

*Background.* Continuous glucose monitoring (CGM) offers an opportunity for patients with diabetes to modify their lifestyle to better manage their condition and for clinicians to provide personalized healthcare and lifestyle advice. However, analytic tools are needed to standardize and analyze the rich data that emerge from CGM devices. This would allow glucotypes of patients to be identified to aid clinical decision-making.*Methods.* In this paper, we develop an analysis pipeline for CGM data and apply it to 148 diabetic patients with a total of 8632 days of follow up. The pipeline projects CGM data to a lower-dimensional space of features representing centrality, spread, size, and duration of glycemic excursions and the circadian cycle. We then use principal components analysis and k-means to cluster patients’ records into one of four glucotypes and analyze cluster membership using multinomial logistic regression.*Results.* Glucotypes differ in the degree of control, amount of time spent in range, and on the presence and timing of hyper- and hypoglycemia. Patients on the program had statistically significant improvements in their glucose levels.*Conclusions.* This pipeline provides a fast automatic function to label raw CGM data without manual input.

## 1. Introduction

Type 2 diabetes mellitus (T2DM) affects one in ten people around the world; this may rise to one in six by 2045 [1]. Patients are at risk of microvascular and macrovascular complications such as stroke and cardiovascular disease [2], and as a result T2DM imposes a serious burden on health systems and economies worldwide. This burden is higher in high income countries [1], but as sedentary lifestyles become common elsewhere, the prevalence is rising in other parts of the world, especially in Asia [3, 4]. In Singapore, a microcosm of South, Southeast, and East Asia, the cost to the workforce is projected to reach close to US$2 billion by 2050 [5].

Lifestyle interventions have been shown to reduce diabetes incidence by 20% among prediabetics and thereby to delay T2DM by 11 years [6]. An even greater incidence reduction of 51% was reported in a 20-year-long study on Chinese participants after dietary and physical activity interventions compared with a control group [7]. A meta-analysis [8] suggested that the diverse effectiveness on glucose reduction in past intervention studies calls for further investigation in heterogeneity of diabetes control. Personalized interventions addressing heterogeneity among patients have proven effective in controlling postmeal glucose in a machine learning study [9], and advances in technology offer hope that personalized treatment using routinely collected health data could revolutionize patients’ outcomes.

Traditional single-point glucose measurements lack the ability to detect glycemic variability and other periodic patterns, which can provide insights for targeted interventions. However, recent technological advances enable patients to overcome this barrier with continuous glucose monitoring (CGM) devices. By extracting glucose variability in moving windows of CGM data, defining glycemic signatures into three groups, and summarizing the entire history by its most frequent patterns, a recent study [10] was able to categorize 57 normoglycemic participants into three “glucotypes.” Furthermore, the frequency of occurrence of high, medium, and low variability in 2-hour window CGM has proven to correlate with clinical measurements, reflecting the underlying physiologic diversity that explains glucose variability.

By enabling drastically more frequent and accessible data than traditional measurements, CGM-based analysis allows physicians to give their patients with diabetes—including type 1 diabetes mellitus and gestational diabetes—detailed, personalized advice on modifying their lifestyle to reduce the incidence of glucose patterns that are known to challenge the body’s long-term ability to regulate blood glucose. In particular, CGM allows quantification of some glucose characteristics that are not available in routine clinical care. Other than blood glucose variability, recent studies recommended clinical targets including the average and time in range [11], while the mean amplitude of glycemic excursions [12] quantifies glucose stability.

In this paper, we develop a classification algorithm to categorize patients wearing CGM devices for intervals of 13 days based on their glycemic characteristics. The data come from patients enrolled in a digital coaching and glucose monitoring program as part of their diabetes outpatient care by NOVI Health, Singapore. There, they receive clinical treatment and personalized lifestyle care-based insights derived from correlations between their lifestyle habits and CGM measurements. We identify four categories or glucotypes, with glycemic features that have relevant clinical implications, using an algorithm that could readily be applied in clinical practice.

The key contribution of this paper is the combination of glycemic characteristics and automated unsupervised classification algorithm towards a more systematic approach to diabetes risk stratification and intervention. This approach allows a standardized set of characteristics to be derived and analyzed from CGM devices, including glycemic stability, excursion, and circadian patterns.

## 2. Methods

### 2.1. Study Design and Participants

Glucose (mmol/L) measurements using continuous glucose monitoring (CGM) were captured roughly once per minute, but the data was available to us in 15-minute intervals using the Abbott FreeStyle consumer version Libre 1 (Abbott Laboratories, Abbott Park, IL, US) flash glucose monitor attached to the patient’s upper arm and extracted and uploaded by the participant using the FreeStyle LibreLink mobile application. Participants were patients of a specialist diabetes outpatient clinic in Singapore (NOVI Health) which offers personalized medical care. Patients signed up for such service and usage of CGM device in intervals of 14 days, the lifespan of the monitoring device. They commonly took a break in between usage intervals, and consequently, CGM of individual patients were of various lengths with intermittent gaps. Besides gaps between 14-day intervals, occasional involuntary detachment of the sensor also contributed to the missing data. The accuracy in the first day is known to be lower than subsequent days [13]; hence, we removed the first day of measurements from each consecutive block of CGM recordings. In addition to CGM measurements, demographic data were recorded at the first visit to clinic, and clinical measurements were recorded at visits to clinic, including mass (kg), height (cm), BMI (kg/m ^2^), waist and hip circumference (cm), waist/hip ratio, HbA1c (%), SBP (mmHg), DBP (mmHg), LDL (mg/dL), HDL (mg/dL), and triglycerides (mg/dL). 

### 2.2. Procedures

**Figure 1:**
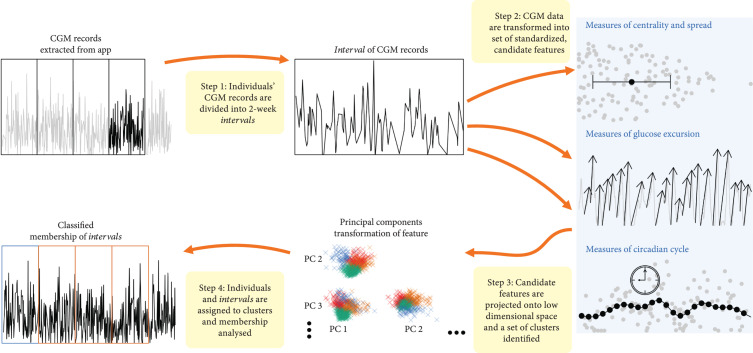
Flowchart of analysis showing the steps taken for classifying CGM. After removing the first day of consecutive CGM records, they were then divided into 13-day intervals and aggregated into features. Based on the feature matrix transformed using principal component analysis, a classification algorithm was applied, thus classifying 13-day intervals of CGM into groups varying in the distribution of features.

The primary objective of the analysis is to group each 13-day interval of CGM records into one of several clusters. This aim is achieved by (i) transforming CGM data into glycemic features and then (ii) fitting the main principal components into a k-means clustering algorithm, as illustrated in Figure 1 and detailed in the Supplemental Material §1. We then (iii) review the labeled CGM intervals and their respective glycemic features using endocrinologic expertise and experience. 

The key step in the analysis pipeline is to transform CGM data into meaningful features for subsequent analysis, thereby overcoming the differences in record lengths and timing that arise in practice. We consider three broad types of feature. First, we consider measures of centrality and spread, including maximum, minimum, mean, standard deviation, mean amplitude of glycemic excursion (MAGE) [12], and the proportion of time spent in abnormal and normal ranges [11] (>13.9, >10, between 3.9 and 10, <3.9, and < 3 mmol/L). Second, we derive measures of glucose excursion, including the average rise and fall, as well as the slopes between valleys and peaks. Last, we measure fluctuations in glucose with the circadian cycle, including the average CGM deviation from the daily mean calculated by collapsing a CGM interval into one day and fitting a spline curve. The identification of excursions is based on the peak detection algorithm Peakdet [14], detailed in the Supplemental Material §3. This algorithm searches for local maximum after local minimum and labels the peak/trough only if the difference from previous trough/peak is beyond a threshold, which we set to ±3 mmol/L based on clinical judgment. 

Full definitions of these features may be found in the supplementary information (available here). 

### 2.3. Statistical Analysis

Based on the feature set, we build an unsupervised classification algorithm to label the 13-day CGM recordings. The feature set is first standardized using the Box-Cox transformation [15] and then transformed into principal components (PCs), from which the main PCs—with at least 80% combined explained variation—are extracted [16]. The main PCs are then used in a k-means classification. Following this, each CGM interval is labeled with a machine-learnt group. By relating these groupings back to glucose features, we *post hoc* assign a clinically meaningful name to the groups. 

To assess the stability of the classification, we investigated pairs of consecutive intervals from the same patient. Clinical measurements were also tested against classification assignment using a multinomial logistic test with generalized estimating equations to account for repeated measurements [17]. 

A threefold cross-validation was used to assess the consistency of clustering. By taking any two folds as the training set and the remaining fold as the test set, we checked the cluster numbers and compared the clustering labels of the three test sets against their original glucotypes. To evaluate the stability between two grouping assignments, the error and accuracy of clustering results were measured by the Adjusted Rand Index [18, 19] and Meila-Heckerman classification accuracy [20]. Adjusted Rand Index calculates the similarity between two unsupervised clustering results based on the pairs of subjects being classified together or separate. Meila-Heckerman classification accuracy first matches up the two sets of labels based on confusion matrix and then calculates the exact accuracy, assuming one set of labels is the “correct” one, similarly to a supervised clustering comparison. 

## 3. Results

We summarize the patient cohort in Table 1. The age of participants ranges from 28 to 81, with 52% less than 40 years old, 21% in their 40s, 13% in their 50s, and 15% more than 60 years old. Most participants are women (68%). Participants have an average BMI of 26 kg/m ^2^ (SD 5 kg/m ^2^), i.e., the majority are overweight or obese under the Singapore classification system [21].

Table 1Demographic distribution of participants. Categorical measurements are summarized in count number per category and corresponding proportion, and numerical measurements are summarized in mean and standard deviation (SD).

**(a):** 

	N *(proportion)*
Diagnosis	
T1DM	6 (5%)
T2DM	51 (46%)
GDM	53 (48%)
Age	
28–39	78 (52%)
40–49	31 (21%)
50–59	19 (13%)
60–81	22 (15%)
Gender	
Female	103 (68%)
Male	48 (32%)

**(b):** 

	*Mean (SD)*
Weight (kg)	72 (15)
Height (cm)	166 (9)
BMI (kg/m ^2^)	26 (5)
Waist (cm)	91 (10)
Hip (cm)	98 (8)
Waist/hip (cm/cm)	1 (0)
HbA1c (%)	7 (1)
SBP (mmHg)	125 (16)
DBP (mmHg)	79 (10)
LDL (mg/dL)	99 (30)
HDL (mg/dL)	57 (17)
Triglycerides (mg/dL)	147 (109)

**Figure 2:**
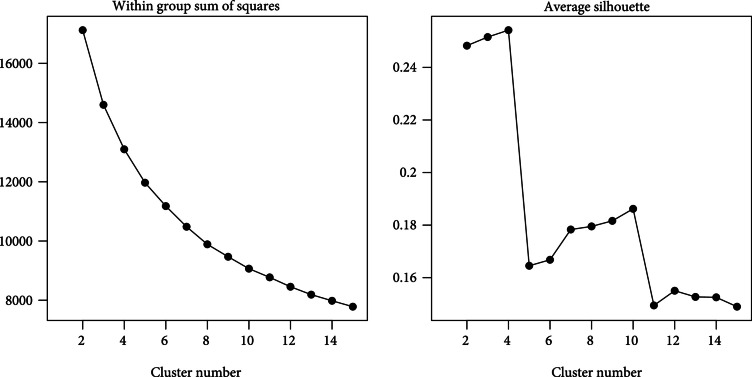
Cluster number detection. Within group sum of squares (a) and average silhouette (b) calculated for cluster numbers from 2 to 15.

**Figure 3:**
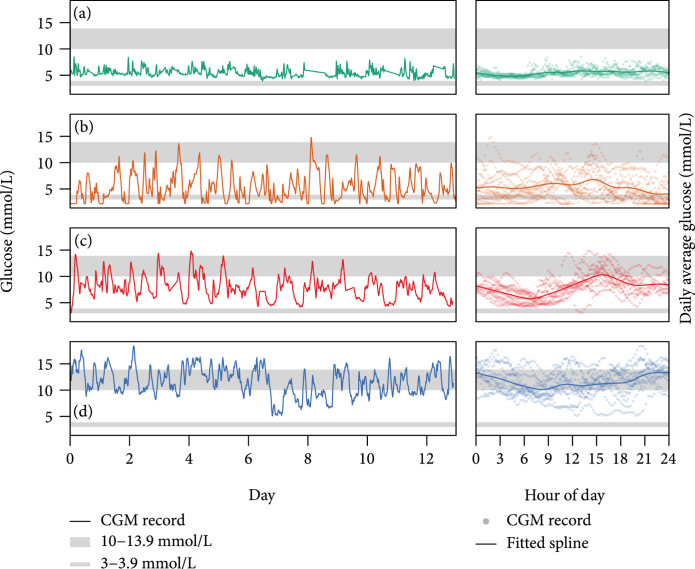
Examples of CGM records. On each row, one 13-day CGM example is picked from each classification groups. (a) The entire CGM records in the 13-day intervals (solid curve); (b) same records mapped into a 24-hour scale based on time of day in timestamp (semitransparent dots), and the spline curves are fitted based on mapped records (smooth curve). Critical cutoffs including 3, 3.9, 10, and 13.9 mmol/L are marked in both panels (gray shaded area).

K-means clustering is applied to the first 7 principal components based on the feature dataset of 664 CGM intervals based on 148 participants. Figure 2 showing within-group sum of squares and average silhouette across cluster numbers suggests the optimal cluster number to be 4. Given the clustering algorithm, a CGM interval is labeled as glucotypes A, B, C, or D. We represent one individual’s interval from each glucotype to illustrate distinct characteristics of the four glucotypes in Figure 3. For example, glucotype A has low and stable glucose levels, and glucotype D has highly fluctuating levels that reflect poor glucose control.

**Figure 4:**
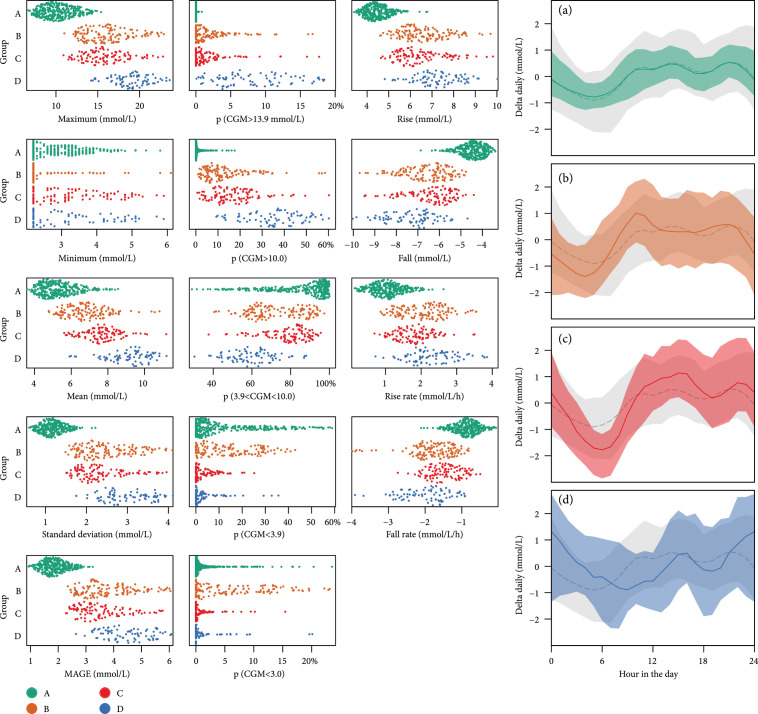
CGM features in four classification groups. First column contains the distribution of features representing centrality and spread. Second column contains the proportion of CGM records in ranges <3, <3.9, in between 3.9 and 10, >10, and>13.9 mmol/L. Third column contains rise and fall features, which are calculated from excursions defined in the Methods section. Last column includes the CGM delta from mean collapsed onto 24 hours, where solid line indicates medium and shadowed area indicates 5th and 95th centiles.

To further illustrate the characteristics distinction, Figure 4 presents all features’ distribution stratified by the four glucotypes. Glucotype A has well-controlled blood glucose with low mean and variability and with slow rises and falls, and not much time spent above critical level (10 or 13.9 mmol/L). Glucotypes B and C are intermediate, with the main differences being a sharp dip in blood glucose levels while sleeping for group C and a greater proportion of time spent below 3 mmol/L for B. Glucotype D has high mean levels, fast rises and falls, the highest variability, and the most time above critical levels. The group characteristics prompted naming them as the following:

(A) Well controlled, with the most time in range (TIR)
(B) Suboptimally controlled, with postprandial hyperglycemia
(C) Suboptimally controlled, with fasting hypoglycemia
(D) Brittlely controlled, with high glucose variability

**Figure 5:**
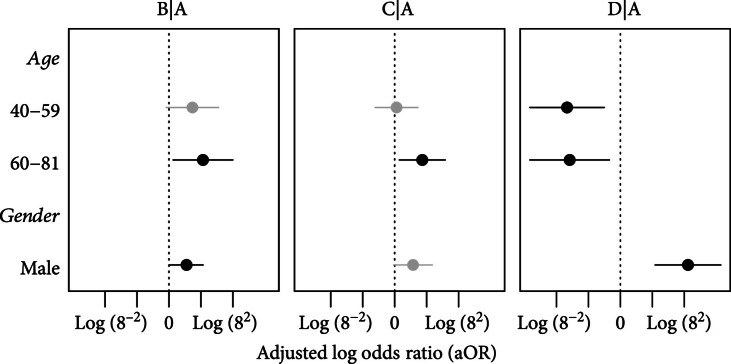
Demographic effects on classification. Adjusted log odds ratio (aOR) of age, gender, and BMI categories for groups B, C, and D with group A as baseline group. Mean aOR is indicated as dots, and confidence interval is indicated as line segment. Gray color indicates insignificant effect, and black indicates significant effect.

The effect of clinical measurements of participants on the group membership is shown in Figure 5, where the adjusted log odds ratio of B, C, and D against A is presented side by side for age and gender. Because the covariance matrix calculations can lead to singularities with large dimensions, we restrict this analysis to the first 10 intervals per participant. The model included age, gender, BMI, and SBP as explanatory variables, but BMI and SBP was omitted from the plot due to its lack of significance in all comparisons. Individuals having older age (60–81yo) were more likely to belong to the group with suboptimal control with postprandial hyperglycemia (B) (aOR 9.14, [95% confidence interval {CI} 1.31, 63.72], respectively). Older patients (60–81yo) have also increased risk of being in the group with suboptimal control with fasting hypoglycemia (C) [6.03, (1.35, 26.83)]. Our middle-aged (40–59yo) and older (60–81yo) patients had a lower risk of being in group D (brittlely controlled) with aOR of 0.03 (0.003, 0.35) and 0.04 (0.003, 0.49). Being male was associated with a greater chance of being in groups B and D with aOR of 3.14 (1.07, 9.21) and 81.31 (9.62, 687.36), respectively.

**Figure 6:**
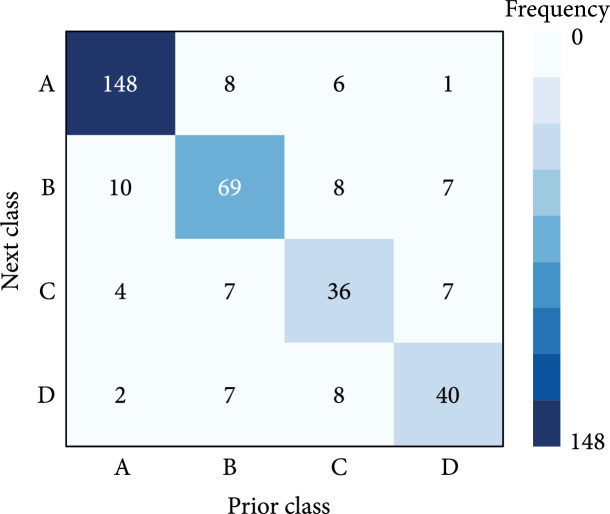
The contingency table of assigned groups for any two consecutive intervals.

**Figure 7:**
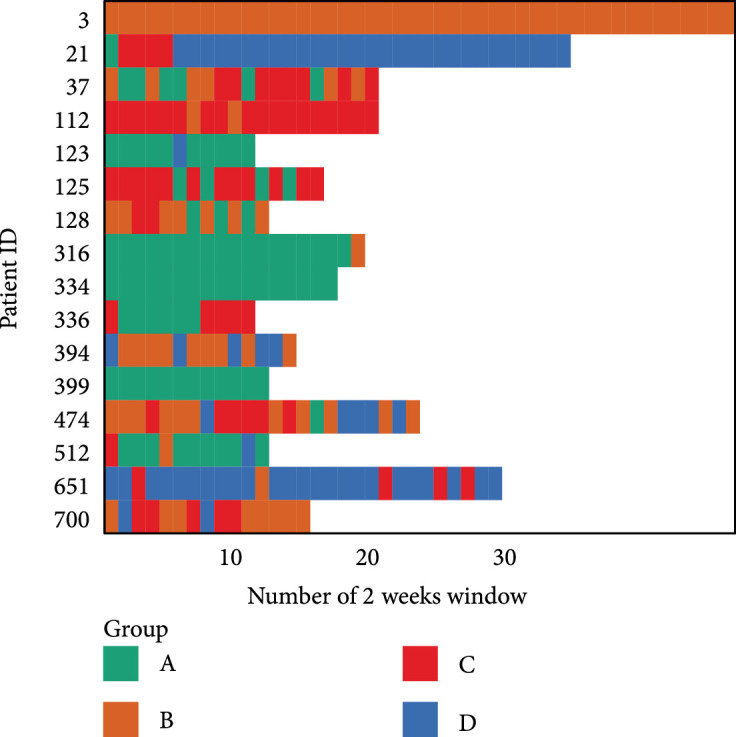
Example of historical assigned groups for a selection of patients with long periods of CGM data.

Figures 6 and 7 show the classifications consistency over time. The contingency table of paired consecutive intervals (Figure 6) shows high consistency between the glucotype classification in two consecutive intervals. Among those (small number of) participants with more than 10 CGM intervals (Figure 7), they are mostly classified as belonging to one or two and usually one group for a prolonged period. Comparing Figures 6 and 7, certain group transition tend to be common. For example, there are noticeable confusions between groups transiting from B to A, C to A, and among B, C, and D, and the pattern can be observed in Figure 7, such as patient ID 128 and 125 corresponding to B-A and C-A, respectively, and patient 474 for B-C, 651 for C-D, and 394 for B-D.

After enrolling in the program, participants after CGM monitoring had a general reduction in glucose levels. A t-test is conducted for the first interval against last interval per participant on their CGM levels, adjusted for circadian cycle average. Among the 80 significant pairs, 51 (64%) participants had significantly higher adjusted CGM in the first interval than the last interval, whereas 29 (36%) had lower adjusted CGM in first interval compared to last interval. There are significantly more participants with reduced CGM after monitoring (P value = 0.02).

Cross validation revealed consistent cluster number choice and assignment based on the average silhouette distance plots. Of the threefold, the Adjusted Rand Index (0.77, 0.79, and 0.90) and Meila-Heckerman classification accuracy (0.81, 0.85, and 0.95) are close to 1, showing high level of consistency with the original clustering result.

## 4. Discussion

In this paper, we have developed a novel protocol for classifying CGM data into four categories using machine learning algorithms, based on carefully calibrated glycemic features. These categories represented different types of glycemic patterns commonly observed in clinical practice among T2DM patients, and the ability to rapidly assign individuals to one such category may form an initial basis for subsequently tailored lifestyle and medical advice. For instance, suboptimally controlled patients with postprandial hyperglycemia would benefit from intensive lifestyle interventions, such as reduction of carbohydrate load at meals and increase postmeal physical activity, as well as medication adjustments to introduce medication with greater postprandial hypoglycemic effects, while those with fasting hypoglycemia would benefit from a medication review to reduce the use of medication together with intensive weight loss. Utilizing CGM in the clinical management of patients with diabetes has been shown to lead to a noticeable improvement in glucose control [22, 23]. The technology has good user acceptability as evinced from the high adherence [23] and repeated use to manage their diet and benefit from its effect on their diabetes. This preliminary study sets the foundation for further work, where the association between the glucotypes, treatment protocols, and clinical outcomes can be studied longitudinally, providing an opportunity to further develop and validate interventions implemented based on the glucotypes.

Recent advances in CGM technology are focused on personalization, such as mobile tracking apps that support physician intervention [24], record diet and fitness activities, and even provide socialization features [25]. The algorithm that we have developed is currently available online (https://sshsphdemos.shinyapps.io/GlucoseAPP/), and the source code of the application is available on GitHub (https://github.com/maoyinan/GlucoseAPP); these allow users to gain a general impression on the overall distribution of both raw and processed data in a structured page and visualize the data of any particular subject in an interactive manner, to help clinicians to identify features of any patient of interest. Publishing the source code allows free adaptation for practitioners in a wider community. Automatization of personalized feedback, akin to new developments such as smart insulin pumps and artificial pancreas systems [26], is an area for future development and in our context could involve automated alerts when high glucose excursions are detected.

The framework we developed regularizes CGM data of different lengths and with the kind of missing data that is common in practice—such as users’ occasional failures to upload records—yet still takes advantage of the rich information embedded in the abundant but intermittent glucose records from CGM, condensing the big data into a lower dimensional set of interpretable features (Supplemental Material §2). Although CGM data is prone to noise and time lag [27, 28], such noise is mitigated through the aggregation step. To prove the claim, we randomly introduced 20% missing data in CGM recordings and compared the features before and after by calculating correlations. Among the 38 features, all are highly correlated (r>.95), supporting the weak impact of missing data on the algorithm. The feature set derivation was partly driven by clinical experience such as the excursion calculator adapted from a local peak detector to compensate for lack of accuracy in traditional glucose excursion measures [27] and partly taken reference from existing literature, such as mean amplitude of glycemic excursions [29] and time in targeted range [11]. The approach was validated by a three-fold cross-validation step and in part through the high consistency in classification of successive intervals of the same patient.

There are however limitations to the work. Our patient pool is relatively young and technologically literate, facilitating their use of the CGM monitors, and thus may not reflect the typical patient profile of those with T2DM, who tend to be older. Patients in this study mostly had T2DM (48%) or gestational diabetes (47%), and the applicability to T1DM is unclear. The ethnicity of patients is mainly Asian, thus contributing to possible bias in the sample of 151 patients. The sample size in patients may seem small, but 670 CGM segments of 14 days were extracted, averaging to 5963 CGM readings per patient, which is not insubstantial, compared to the similar glucotypes classification study based solely on glucose variability, where 2-4 weeks CGM readings were extracted from a subject pool of 57 [10]. The decision to have four glucotypes was based on statistical criteria, and although the categories comported with clinical experience, it is possible that in larger pools of patients, additional, distinct categories might be observed. Finally, although we observed a general improvement in blood glucose measurements after starting treatment, the long-term malleability of glucotype membership is unclear and should be the target of further study.

## 5. Conclusions

In conclusion, this study gives a new approach in classifying CGM data, through an informative collection of glycemic features. The automated algorithm functions as a complimentary tool in clinical setting to assist personalized glucose management in both patients with diabetes and prediabetes. Integrated with other biometrics data, the categorization method can be utilized to guide targeted interventions among patients with diabetes.

## Data Availability

Data are available on reasonable request.

## Supplementary Materials

Supplementary Materials 1 Study design: The analysis flowchart in Figure 1 summarizes the main study plan. This section aims to supplement the flowchart by providing a comprehensive description of the data preprocessing and the analysis pipeline. We summarize the study design by the following parts: the dataset description, the pre-processing steps, and the statistical analysis. 2 List of glycemic features: The mathematical notations of the 38 CGM features are detailed in this section. Records of 13-day interval CGM data are aggregated into 38 features according to three aspects: (1–10) centrality and spread; (11–14) measures of excursion; and (15–38) average deviation from mean values over the circadian cycle. 3 Procedure to label local extremes: To prepare for calculating the measures of excursion in the previous section, this section describes the algorithm to search for local peaks and valleys in the form of sudo code, as originally outlined by peakdet [14].
