# Supplementary material for: Stratification of Patients with Diabetes Using Continuous Glucose Monitoring Profiles and Machine Learning
Source: Health Data Sci. 2022 Apr 27;2022:9892340. doi: 10.34133/2022/9892340 (PMC10880155; doi:10.34133/2022/9892340)
Supplement: Supplementary Materials — 1 Study design: The analysis flowchart in Figure 1 summarizes the main study plan. This section aims to supplement the flowchart by providing a comprehensive description of the data preprocessing and the analysis pipeline. We summarize the study design by the following parts: the dataset description, the pre-processing steps, and the statistical analysis. 2 List of glycemic features: The mathematical notations of the 38 CGM features are detailed in this section. Records of 13-day interval CGM data are aggregated into 38 features according to three aspects: (1–10) centrality and spread; (11–14) measures of excursion; and (15–38) average deviation from mean values over the circadian cycle. 3 Procedure to label local extremes: To prepare for calculating the measures of excursion in the previous section, this section describes the algorithm to search for local peaks and valleys in the form of sudo code, as originally outlined by peakdet [14]. [file 9892340.f1.docx]

**Supplementary Material**

**1 Study design**

The analysis flowchart in Figure 1 summarizes the main study plan. This section aims to supplement the flowchart by providing a comprehensive description of the data preprocessing and the analysis pipeline. We summarize the study design by the following parts: the dataset description, the pre-processing steps, and the statistical analysis.

**1.1 Dataset description**

The anonymized raw dataset is comprised of three data matrices. Each is identifiable by patients’ unique ID numbers, but they contain different sets of information. The records were captured only once in the demographic dataset, but repeatedly for the clinical measurement dataset and the CGM dataset at different frequencies. The linkage to the same unique identifiers facilitates merging the three datasets into one large dataset. All three datasets have missing values.

**Demographic dataset**

Each patient was recorded once at the first visit to the clinic for the demographic, lifestyle and medical history features: diagnoses, date of birth, gender, ethnicity, smoking status, alcohol consumption (units per week), and age. Only gender and age were used in analysis; the other variables were omitted due to a degree of incompleteness.

**Clinical measurement dataset**

The clinical measurements were recorded at patients’ visits to clinic, including mass (kg), height (cm), BMI (kg/m^2^), waist and hip circumference (cm), waist/hip ratio, HbA1c (%), SBP (mmHg), DBP (mmHg), LDL (mg/dL), HDL (mg/dL) and triglycerides (mg/dL). At the subject level, the last observation carried forward method was adopted for missing data imputation, namely, the most recent record before the missing data was used to fill the gaps.

**CGM dataset**

Captured from a continuous glucose monitoring device, the records constitute a time-dependent longitudinal dataset. The interval between successive records is roughly 15 minutes, but the data are not strictly equally spaced in time. Patients subscribed to CGM monitoring for a multiple of 14 days’ period. Frequent gaps exist in between a patient’s consecutive subscriptions. The CGM dataset is merged with the clinical measurement dataset by exact matching patient ID and fuzzy matching the most recent clinical visit date.

**1.2 Pre-processing in the subject level**

After preparing the completed and merged dataset, we processed the CGM records for statistical analysis. It is structured as a long matrix with patient ID, CGM recording time, demographic features, clinical measurements as the column variables. The CGM records are irregularly spaced in time, and nonequal in length among patients. The following steps are taken to tackle the complex data structure.

1. Iterating through patients, we split the time-dependent CGM records into separate blocks whenever the gaps before or after are longer than 24 hours.
2. The first 24 hours of each block of consecutive CGM records are discarded due to inaccuracy^1^.
3. We then further divide the CGM blocks into intervals of 13-days and label them sequentially.
4. The resulted 13-day CGM intervals are pooled and treated as input data, to be aggregated into features for statistical analysis.
5. We then iterate through intervals, and calculate features based on the algorithm described in Supplementary Material §2. The above steps produce a feature matrix $F$ with rows corresponding to intervals and columns to features.

**1.3 Statistical analysis**

With the aim to stratify CGM 13-day intervals into interpretable diabetic profiles, we take additional processing steps and conduct relevant statistical inferences to classify, interpret, check data sanity, and test hypotheses. These are:

1. We normalize the features matrix $F$into $F_{1}$ by applying the Box-Cox transformation to the columns of $F$.
2. We then apply principal component analysis to transform the normalised feature matrix $F_{1}$ into a principle components matrix $F_{2}$ with a smaller dimension. The number of principle components is chosen by the minimum number at which the combined explained variance meets at least 80% of total explained variance.
3. We then apply the K means clustering method on the principal component matrix $F_{2}$. This step automatically groups all the 13-day intervals into a number of clusters, with the number being selected optimally via maximising the between group distance, and minimising the within group distance.
4. We then review CGM records in 13-day intervals with the assigned group labels manually, and combine these with clinical judgement to summarise the unique CGM patterns differing among the stratified groups.
5. We then assess the stability of the stratification by tabulating the consecutive CGM groups, which is expected to concentrate at diagonal in the contingency table, namely that successive 13-day interval be labelled as belonging to the same group. We also manually check the stratification of the entire CGM history of patients to ensure that the classification accords with our expectations.
6. Finally we investigate the effects of clinical measurements: age, gender, BMI and SBP on stratification by conducting a multinomial logit test. The continuous variables are discretized to account for nonlinearity. Age is grouped as three categories: <40, 40-59, 60–81yo, BMI as <27.5 and 27.5+, and SBP as <140 and 140+.

**2 List of glycemic features**

Records of 13 days’ interval CGM data are aggregated into 38 features according to three aspects: (1–10) centrality and spread; (11–14) measures of excursion; (15–38) average deviation from mean values over the circadian cycle. We denote records (mmol/L) in interval $M$ of 13 days of a patient as $\left\{ y_{i} | i=1,\ldots,n_{M} \right\}$ at time points in unit of hour $\left\{ t_{i} | i=1,\ldots,n_{M} \right\}$ with $n_{M}$ as the total number of observations in the interval $M$. Note that CGM records are of unequal lengths among CGM intervals, namely, $n_{M}$ differs among $M$. Glycemic features of an interval $M$ are calculated as described below. To reduce notation complexity, we simplify $n_{M}$ by $n$ since the operations are conducted on each interval independently of other intervals.

1. Maximum (mmol/L) ${=max}_{\{i=1,...,n\}} y_{i}$
2. Minimum (mmol/L) ${=min}_{\{i=1,...,n\}} y_{i}$
3. Mean (mmol/L) $=\sum_{i=1}^{n} y_{i} /n:=\bar{y}$
4. Standard deviation (mmol/L) $=\sqrt{{\sum_{i=1}^{n} {(y}_{i}-\bar{y})}^{2}/(n-1)}:= sd(y)$
5. MAGE (mmol/L) $=\sum_{j=1}^{m} \left| y_{i}- \bar{y} \right|\cdot I\left( \left| y_{i}- \bar{y} \right|>\left. sd(y \right) \right)/m$ where $m=\sum_{i=1}^{n} I\left( \left| y_{i}- \bar{y} \right|>\left. sd(y \right) \right)$, and $I(\cdot)$ is the indicator function.
6. p(CGM>13.9) $=\sum_{i=1}^{n} I\left( y_{i}>13.9 \right)/n$
7. p(CGM>10.0) $=\sum_{i=1}^{n} I\left( y_{i}>10 \right) /n$
8. p(3.9<CGM<10.0) $=\sum_{i=1}^{n} I\left( {3.9<y}_{i}<10 \right)/n$
9. p(CGM<3.9) $=\sum_{i=1}^{n} I\left( y_{i}<3.9 \right) /n$
10. p(CGM<3.0) $=\sum_{i=1}^{n} I\left( y_{i}<3 \right) /n$
11. Rise (mmol/L) $=\sum_{k=1}^{K-1} ( P_{k+1}-V_{k})/(K-1)$ where $P_{k}$ and $V_{k}$ are defined based on the search algorithm in Supplemental Material §3.
12. Fall (mmol/L) $=\sum_{k=1}^{K} {(P}_{k} -V_{k})/K$
13. Rise rate (mmol/L/h) $=\sum_{k=1}^{K-1} \frac{P_{k+1}- V_{k}}{t_{P,k+1}-t_{V,k}}/(K-1)$
14. Fall rate (mmol/L/h) $=\sum_{k=1}^{K} \frac{P_{k}-V_{k}}{t_{P,k}-t_{V,k}}/K$
    1. Delta (mmol/L) at $h$ o’clock $=f(h|y_{i},t_{i} \% 24, i=1,...,n)-\bar{y}$ where $h=0,...,23$ (midnight being 0) and $f(\cdot)$ denotes the fitted value at time $h$ given the spline curve with record vector $\left\{ y_{i} | i=1,\ldots,n \right\}$ at time vector {$t_{i}\%24, i=1,...,n$}, transformed from ${\{t}_{i}, i=1,...,n$} by collapsing into circadian 24 hours of time. Here, the notation $\%$ denotes modulus operation.

**3 Procedure to label local extremes**

To prepare for calculating the measures of excursion, first we denote local peaks and valleys as $\left\{ P_{k}, V_{k} | k=1,\ldots,K \right\}$ at time points $\left\{ t_{P,k},t_{V,k} | k=1,\ldots,K \right\}$, where $K\in\{1,\ldots,\left\lfloor n/2 \right\rfloor\}$ in which $\left\lfloor\cdot\right\rfloor$ is the floor function. They are searched in succession from candidates $\left\{ y_{i} | i=1,\ldots,n \right\}$, such that they are local extremes and the distance between consecutive peaks and valleys, $P_{k}-V_{k}$ and $P_{k+1}-V_{k}$ respectively, is no less than a threshold $D$ arbitrarily set to 3 (mmol/L). The search algorithm is summarized by the following steps, originally outlined by peakdet^2^.

1. Initialize $i=1,k=1,X_{1}=\infty,X_{2}=-\infty,t_{1}=t_{2}=0$.
2. Let $X=y_{i}$
3. If $X>X_{2}$ then $X_{2}=X, t_{2}=t_{i}$.
4. If $X<X_{1}$ then $X_{1}=X, t_{1}=t_{i}$.
5. Search for peak $P_{k}$: if $X<X_{2}-D$ then set
   1. $P_{k}=X_{2},t_{P,k}=t_{2}$ and
   2. $X_{1}=X,t_{1}=t_{i}$.
6. Increment $i$ by one.
7. Repeat step 2) to 6) until $P_{k}$ is found
8. Repeat step 2) and 4)
9. Search for valley $V_{k}$: if $X>X_{1}+D$ then
   1. $V_{k}=X_{1}, t_{V,k}=t_{1}$
   2. $X_{2}=X, t_{2}=t_{i}$
10. Increment $i$ by one.
11. Repeat step 8) to 10) until $V_{k}$ is found
12. Increment $k$ by one.
13. Go back to step 2) until $i=n$.

**References in supplement**

1. Bailey T, Bode BW, Christiansen MP, Klaff LJ, Alva S. The Performance and Usability of a Factory-Calibrated Flash Glucose Monitoring System. *Diabetes Technol Ther*. 2015;17(11):787-794. doi:10.1089/dia.2014.0378

2. peakdet: Peak detection using MATLAB (non-derivative local extremum, maximum, minimum). Accessed April 29, 2021. http://www.billauer.co.il/peakdet.html
